# Positivity in Younger and in Older Age: Associations With Future Time Perspective and Socioemotional Functioning

**DOI:** 10.3389/fpsyg.2020.567133

**Published:** 2020-11-17

**Authors:** Miray Erbey, Josefin Roebbig, Anahit Babayan, Deniz Kumral, Janis Reinelt, Andrea M. F. Reiter, Lina Schaare, Marie Uhlig, Till Nierhaus, Elke Van der Meer, Michael Gaebler, Arno Villringer

**Affiliations:** ^1^Department of Neurology, Max Planck Institute for Human Cognitive and Brain Sciences, Leipzig, Germany; ^2^MindBrainBody Institute at the Berlin School of Mind and Brain, Humboldt-Universität zu Berlin, Berlin, Germany; ^3^International Max Planck Research School on the Life Course, Max Planck Institute for Human Development, Berlin, Germany; ^4^Lifespan Developmental Neuroscience, Technische Universität Dresden, Dresden, Germany; ^5^International Max Planck Research School NeuroCom, Leipzig, Germany; ^6^Department of Education and Psychology, Center for Cognitive Neuroscience Berlin, Freie Universität Berlin, Berlin, Germany; ^7^Department of Psychology, Humboldt-Universität zu Berlin, Berlin, Germany; ^8^Department of Cognitive Neurology, Leipzig University Hospital, Leipzig, Germany

**Keywords:** positivity effect, positivity bias, socioemotional selectivity theory, future time perspective, well-being, aging, socioemotional functioning, worry

## Abstract

Aging has been associated with a motivational shift to positive over negative information (i.e., positivity effect), which is often explained by a limited future time perspective (FTP) within the framework of socioemotional selectivity theory (SST). However, whether a limited FTP functions similarly in younger and older adults, and whether inter-individual differences in socioemotional functioning are similarly associated with preference for positive information (i.e., positivity) is still not clear. We investigated younger (20–35 years, *N* = 73) and older (60–75 years, *N* = 56) adults’ gaze preferences on pairs of happy, angry, sad, and neutral faces using an eye-tracking system. We additionally assessed several parameters potentially underlying inter-individual differences in emotion processing such as FTP, stress, cognitive functioning, social support, emotion regulation, and well-being. While we found no age-related differences in positivity when the entire trial duration was considered, older adults showed longer fixations on the more positive face in later stages of processing (i.e., *positivity shifts*). This allocation of resources toward more positive stimuli might serve an emotion regulatory purpose and seems consistent with the SST. However, our findings suggest that age moderates the relationship between FTP and positivity shifts, such that the relationship between FTP and positivity preferences was negative in older, and positive in younger adults, potentially stemming from an age-related differential meaning of the FTP construct across age. Furthermore, our exploratory analyses showed that along with the age and FTP interaction, lower levels of worry also played a significant role in positivity shifts. We conclude that positivity effects cannot be solely explained by aging, or the associated reduced FTP *per se*, but is rather determined by a complex interplay of psychosocial and emotional features.

## Introduction

Late adulthood has been characterized in several studies by increased emotional stability and emotional well-being in comparison to early adulthood (Carstensen et al., 2000, 2011). For instance, in a cross-sectional study of younger and older adults, the oldest age group (45–75 years old) reported having the most positive, along with the least negative affect (Mroczek and Kolarz, 1998). Furthermore, in several experimental settings, older adults were shown to display more attention and memory for positive and less for negative stimuli compared to younger adults (Mather and Carstensen, 2003, 2005; Knight et al., 2007; Allard and Isaacowitz, 2008). This age-group difference in greater preference for positive over negative information in information processing has been named the *positivity effect*, and it is assumed to play an important role in emotional functioning and well-being of the elderly (Carstensen and Mikels, 2005; Reed and Carstensen, 2012).

While the positivity effect has been extensively studied and replicated in many studies, and has been recognized as an important cornerstone to explain emotional development across the life span, some questions continue to be debated: These concern (i) the reliability of the effect and the conditions for its occurrence, (ii) its grounding in theory, and (iii) potential factors modulating increased attention to positive information.

Regarding the reliability of the positivity effect, several authors have pointed to a lack of consistency (Grühn et al., 2005). Mixed findings were mostly attributed to differences in study paradigms, suggesting that the positivity effect is more likely to emerge when participants are free of explicit cognitive demands and can allocate their attention to stimuli at their leisure (Reed et al., 2014). Attentional deployment, which denotes diverting attention from or reallocating attention to specific aspects of emotional stimuli, has been suggested to be an emotion regulation strategy according to theories on emotion regulation (Gross, 1999). Previous studies have indicated that attentional deployment is more often used by older rather than by younger adults as an emotion regulatory tool (Lohani and Isaacowitz, 2014). Eye tracking allows direct and continuous assessments of fixations, and is a useful method for a fine-grained measure of early and sustained attention (Vazquez et al., 2016). For instance, studies using eye tracking have shown that older adults display positivity especially in later stages of processing (e.g., Isaacowitz et al., 2009; Lee and Knight, 2009). Delayed onset of positivity (i.e., shifting attention toward positive and away from negative stimuli) would be consistent with an allocation of resources serving an emotion regulatory purpose, supporting the thesis that older adults with higher executive functioning show greater positivity in information processing (Mather and Knight, 2005).

Moreover, studies using eye tracking have shown that older adults tend to focus on mouth regions, whereas younger adults spend more time looking at the eye regions, which is more informative in recognizing negative expressions (Sullivan et al., 2007). In line with this, older adults were shown to perform worse at recognizing emotions such as anger, sadness and fear, but show no consistent difficulties in recognizing emotions such as happiness, surprise and disgust (Isaacowitz et al., 2007). This brings us to question whether the positivity effect observed in older age arise from a differentiation in focusing on different regions of faces. Investigating gaze preferences on face stimuli might help answer whether older adults who look more at the mouth regions are the ones who show more positivity effect in general.

Regarding the conceptual grounding, the positivity effect is often explained in the framework of socioemotional selectivity theory (SST; Carstensen et al., 1999), a life-span theory which posits that human priorities shift with changes in perceived time horizons. When people perceive their remaining lifetime as limited, they tend to prioritize present goals such as optimizing well-being over future-oriented goals such as acquiring knowledge (Charles and Carstensen, 2010). Favoring positive over negative stimuli in information processing has been suggested to reflect a goal-directed, top-down regulatory effort for optimizing emotional experience (Reed and Carstensen, 2012). Consistent with the proposal that a limited future time perspective (FTP) drives the positivity effect, Kellough and Knight (2012) found that imagining an open-ended FTP reduced positivity in older adults’ perception of emotions. Interestingly, however, imagining a limited FTP did not lead to a change in younger adults’ interpretations of emotions in this study. This raises the question whether the theory only consistently applies to older adults. According to the SST, it is the perceived remaining life time—rather than the age *per se*—that drives the differences in motivation for optimizing emotional experience. However, perceiving future life time as limited might have different meanings for different age groups. The self-report FTP questionnaire (Carstensen and Lang, 1996) involves items such as “Many opportunities lie ahead of me in the future.” A person’s only weak support for such a statement might indicate a more realistic (for an older person), or a pessimistic attitude (for a younger person), depending on one’s age. The idea that perceiving future life time as limited might have different meanings for different age groups is not explicitly discussed in positivity effect research (within the framework of SST), although this might be a potential qualifier to the theory (Grühn et al., 2016). Although FTP has been the most relevant construct in explaining age differences in the positivity effect, questions such as whether a top-down, motivated attention to positive information would be similarly linked to a limited FTP in younger and in older adults, and whether any potential differences can be explained by differences in socioemotional functioning remain open.

Finally, based on the premise that age alone is not a sufficient causal factor for psychological processes (e.g., Baltes and Lang, 1997; Baltes et al., 2006), we reasoned that inter-individual differences in abilities, resources, or emotional states might contribute to an individual’s top-down motivation for positive stimuli. In light of previous research, we examined several socioemotional factors alongside age that might be relevant for the positivity effect. One such factor that has been implicated to influence top-down positivity in information processing is cognitive functioning (Mather and Knight, 2005; Reed and Carstensen, 2012). Another potential factor might be *stress*. Compared to younger adults, older adults experience fewer interpersonal tensions (Birditt et al., 2005), report fewer daily stressors (Charles et al., 2010), and avoid negative stimuli during acute stress (Ellenbogen et al., 2002). Furthermore, studies on emotion regulation have shown that older adults are more likely to use distraction (Scheibe et al., 2015), and avoid distressing situations or appraise unavoidable situations as less severe (Stawski et al., 2008). Increased motivation for positive information, and avoiding stressful/negative situations might be related to the same underlying emotion regulatory mechanism. However, although the positivity effect is assumed to have an emotion regulatory role, how individual differences in emotion regulation and chronic stress relates to differences in positivity in emotion processing is not known. Availability of *social support* might also be a relevant factor for positivity. Focusing primarily on positive information would not be an evolutionarily adaptive mechanism, as detecting negative information is necessary to deal with potential threat or danger (LeDoux, 1995). Older adults do not show positivity in situations where avoiding the negative might have detrimental effects on their well-being (English, 2012; Reed and Carstensen, 2012). Negative stimuli or outcomes can more easily be dealt with in a group or with *perceived* assistance from one’s peers, and lack of social support might make negative effects more apparent for some older adults (Russell and Cutrona, 1991; Dalgard et al., 1995). Therefore, a person who lacks social support might be more primed than others to focus more on the negative (in order to avoid its potential negative consequences). Thus, the amount of social support that older adults receive may modulate their focus on positive or negative information. Finally, although increased attention to positive information has been commonly linked to everyday functioning and positive outcomes, i.e., *well-being* (Reed and Carstensen, 2012), whether well-being is a significant predictor of motivated, top-down attention to positive information is not clear.

### Present Study

Based on the above considerations, we pursued three main aims in this study: (1) to identify whether there are age-related time-course differences in gaze patterns (positivity shift) during observation of pairs of “more positive vs. more negative” face pictures, (2) the role of FTP in positivity shifts, and whether FTP is similarly related to socioemotional functioning in younger and older adults, (3) how inter-individual differences in socioemotional functioning alongside age relate to positivity shifts.

To pursue these aims, we used a free viewing task where younger and older adults’ gaze patterns on pairs of happy, angry, sad, and neutral face stimuli were recorded using an eye-tracking system. We followed Reed and Carstensen’s (2012) definition that “positive processing preference can result from heightened processing of positive *and/or* reduced processing of negative information” (p. 2), and quantified positivity by assessing the relative fixation durations on the “more positive” side of the five contrasts (happy vs. sad, happy vs. angry, happy vs. neutral, neutral vs. angry, and neutral vs. sad). Taking into account previous findings on delayed occurrence of positivity, we tested whether age differences in positivity preferences differentially emerge in early and late trial periods (i.e., positivity shifts), as opposed to when the entire viewing period (i.e., positivity bias^1^) was considered. We hypothesized that older adults would show greater positivity for the entire trial durations, and especially greater *positivity shifts* than younger adults.

In a *post-hoc* analysis,^2^ we also examined whether younger and older adults show a differential preference for sad and angry faces. Further analyses on gaze preferences examined whether there are age-related differences in focusing on different regions of faces. We hypothesized that increased positivity in older age might be related to a preference for looking at different regions of faces.

We assessed perceived FTP in all participants. We hypothesized that the relationship between FTP and the positivity shift would differ as a function of age, such that a limited FTP would be associated with greater positivity shifts in older, but not in younger adults.

Regarding inter-individual differences, we further hypothesized: (1) executive functioning, increased positive emotion regulation, and higher subjective well-being would be positively, whereas factors related to stress would be negatively associated with positivity shifts, (2) a higher social support would be associated with greater positivity shifts especially in older adults, (3) the relationship between FTP and other socioemotional functioning measures, such as well-being and stress, would be different across the age groups.

## Materials and Methods

The current study was planned and carried out as part of a larger cross-sectional study, the “Leipzig study for mind-body-emotion interactions” (LEMON), established at the Max Planck Institute for Human Cognitive and Brain Sciences (MPI CBS) in Leipzig (for details, see Babayan et al., 2019).

### Participants

For the LEMON study, healthy participants between the age of 20–35 and 59–77 were recruited through the MPI CBS’s participant database, online advertisements, and flyers distributed in public spaces and at the University of Leipzig, Germany. Participants were all Caucasian and native German speakers. Exclusion criteria comprised a diagnosis of cardiovascular (e.g., hypertension, heart attack), neurological (e.g., multiple sclerosis, stroke), psychiatric (with inpatient treatment > 2 weeks within the last 10 years; e.g., psychosis, PTSD; with inpatient treatment > 2 weeks within the last 10 years) or any other malignant disease (e.g., cancer); consumption of psychoactive drugs (e.g., MDMA, THC) or excessive alcohol; intake of centrally active medications (amber, beta- or alpha blocker, cortisol, any chemotherapeutic, or psychopharmacological medication); and standard MRI exclusion criteria (e.g., metallic implants, tattoos, pregnancy, claustrophobia). Current or past study of psychology and previous participation in a scientific study within the last 10 years were also among the exclusion criteria (see Babayan et al., 2019).

Inclusion to the study was performed in two steps: people were prescreened via telephone, during which potential exclusion criteria were assessed. Individuals who did not meet any exclusion criterion were invited to MPI CBS and were individually interviewed by the study physician to ensure that none of the exclusion criteria were met. For this study, after the exclusion criteria were applied to the full sample of 170 participants who completed the task, 12 participants were excluded due to positive drug tests and 4 participants were excluded due to a current alcohol or substance use disorder diagnosed with the Structured Clinical Interview for the *DSM-IV* (SCID-IV; First et al., 1996) measurement, leaving 154 people for the analyses. The study was carried out in accordance with the Declaration of Helsinki and the study protocol was approved by the ethics committee at the medical faculty of the University of Leipzig (reference number 154/13-ff). All participants signed a consent form according to the protocols approved by the ethics committee of the Medical Faculty at the University of Leipzig.

After eye-tracker data from 25 participants (13 older, 12 younger adults) were excluded from the analysis,^3^ 129 participants remained for the analysis of this study. 73 were younger (*M*_age_ = 24.49, age range: 20–35 years, 40 female) and 56 were older (*M*_age_ = 67.33, age range: 60–75 years, 35 female) adults.

Older adults reported having longer education (in years) than younger adults. Younger adults reported higher levels of worry, using more positive emotion regulation strategies, and a more open-ended FTP than older adults, and they scored higher on cognitive functioning than older adults. There were no significant age-related differences on well-being, lack of resources, or in social support measures (see Table 1). Correlations between our study variables for each group can be seen in Table 2.

**TABLE 1:** Demographic information and emotional and cognitive functioning measures.

	Younger adults (*n* = 85)		Older adults (*n* = 69)		Group comparisons	
	*M*	*SD*	*M*	*SD*	*χ2*	*p*
Age	24.49	3.06	67.33	4.67		
Sex (female:male)	45:40		35:34		0.10	0.747

	***M***	***SD***	***M***	***SD***	***t***	***p***

Education (in years)	13.63	1.81	14.69	1.88	–3.45	<0.001
Well-being	34.21	5.75	33.73	5.62	1.14	0.253
Stress due to lack of resources	1.29	1.34	–1.58	0.96	0.14	0.884
Burden	0.14	1.30	–1.82	0.87	1.77	0.073
Worry	0.24	1.18	–3.03	0.81	3.24	0.001
Positive emotion regulation	1.39	0.83	–1.70	0.97	2.08	0.032
Satisfaction with social support	7.14	2.00	7.46	1.79	–1.00	0.311
Practical social support	22.40	3.29	22.85	3.02	–0.85	0.392
Future time perspective	52.08	8.01	37.81	9.49	9.31	<0.001
Cognitive functioning	0.61	0.47	–0.75	0.78	13.25	<0.001

**TABLE 2:** Correlations between key predictors in younger and in older adults.

Younger adults	1	2	3	4	5	6	7	8	9
1. PS	–								
2. SLR	–0.08	–							
3. Burden	0.04	−0.49**	–						
4. Worry	−0.26*	−0.32**	−0.24*	–					
5. FTP	0.42***	–0.13	0.14	−0.31*	–				
6. SSS	0.08	−0.51***	0.21	–0.05	0.24**	–			
7. PSS	–0.02	−0.38***	0.25*	–0.01	0.37**	0.46***	–		
8. PER	–0.07	–0.15	0.22*	–0.12	0.28*	0.12	0.17	–	
9. Cognition	0.09	–0.11	–0.02	0.13	0.09	0.13	0.30**	–0.02	–
10. Well-being	0.20	−0.47***	0.42***	−0.31**	0.47***	0.47***	0.42***	0.36***	0.20

**Older adults**	1								

1. PS	–								
2. SLR	0.19	–							
3. Burden	–0.02	−0.32**	–						
4. Worry	−0.26*	−0.47*	–0.04	–					
5. FTP	−0.27*	−0.36**	0.26*	0.11	–				
6. SSS	0.17	–0.01	–0.08	–0.16	–0.04	–			
7. PSS	0.12	−0.35**	0.15	–0.02	0.25*	0.37**	–		
8. PER	0.15	0.09	0.26*	−0.24*	0.04	0.25*	0.32**	–	
9. Cognition	–0.06	–0.04	0.07	–0.17	–0.04	0.16	0.06	0.04	–
10. Well-being	–0.01	−0.44***	0.04	–0.01	0.16	0.33**	0.50***	0.11	0.17

PS, Positivity shift; SLR, Stress due to lack of resources; FTP, Future time perspective; SSS, Satisfaction with social support; PSS, Practical social support; PER, Positive emotion regulation. p < 0.001 “***,” p < 0.01 “**,” p < 0.05 “*.”

### Sample Size and Power

We performed the power analysis of our study on the basis of data from Isaacowitz et al., 2009; *N* = 79). They found that older adults’ positivity preferences emerged only 500 ms after the stimulus onset and increased linearly over time (effect size η^2^ = 0.06 for Age Group × Emotion × Time Course). We used G^∗^Power 3.1 (Faul et al., 2009) to perform the power analyses. With an alpha level of 0.05, and power of 0.95, the projected sample size that is needed to detect an effect size of *f*^2^ = 0.25 is 54 participants for a repeated-measures analysis of variance (ANOVA) with a within and between subjects interaction. Moreover, in order to test our hypothesis for the FTP × Age Group interaction, we additionally performed sensitivity power analysis using “Linear Multiple Regression: Fixed Model, *R*^2^ increase,” and calculated the projected sample size to detect small (*f*^2^ = 0.02), medium (*f*^2^ = 0.15), and large (*f*^2^ = 0.35) effect sizes. We specified the number of tested predictors as 1, and number of total predictors as 3 (i.e., FTP, Age Group, and a two-way interaction that is between FTP × Age Group). The power analysis revealed that the projected sample size needed to be 635 to detect a small effect, 129 to detect a medium effect, and 57 to detect a large effect. Taken together, these analyses show that our sample size of 129 should be adequate to examine the main objectives of this study, as long as the FTP × Age Group interaction *f*^2^ is larger than 0.15.

### Measures

#### Well-Being

To have a general measure of subjective well-being, we used the “well-being” subscale of the *Trait Emotional Intelligence Questionnaire* (TEIQue-SF; Petrides and Furnham, 2006; German adaptation by Jacobs et al., 2015), which covers three facets of well-being: Self-esteem, Trait-happiness, and Trait-optimism. This factor has also been labeled as “trait positivity” and has been shown to be tightly associated with life satisfaction (Siegling et al., 2015). Items on this scale include statements such as “On the whole, I’m pleased with my life,” and are answered on a 7-point Likert-type scale from 1 (*completely disagree*) to 7 (*completely agree*). Cronbach’s alpha for this subscale was 0.80.

#### Stress

Perceived stress levels were measured using two questionnaires. The *Trierer Inventar zum Chronischen Stress* (TICS; Schulz and Schlotz, 1999), which assesses chronic stress over the last 3 months with nine subscales (Work Overload, Social Overload, Pressure to Perform, Work Discontent, Excessive Demands at Work, Lack of Social Recognition, Social Tension, Social Isolation, and Chronic Worrying), and the *Perceived Stress Questionnaire* (PSQ; Cohen et al., 1983). The PSQ assesses the perception, appraisal, and processing of stressors (“global stress”) from the previous 2 years and includes four subscales: Worries, Tension, Joy, and Demands.

As the TICS and PSQ assess related constructs, a maximum likelihood factor analysis^4^ with promax oblique rotation on a total of 13 subscales was used in the testing sample (*N* = 129). Cronbach’s alpha for the 13 subscales was 0.67. The factor analysis extracted three factors (based on Complexity 1 and Complexity 2 Criteria of *Very Simple Structure*, VSS; Revelle and Rocklin, 1979). Items with salient loadings on Factor 1 corresponded to lack of satisfaction with one’s job and one’s social conditions; items with high loadings on Factor 2 corresponded to subscales related to external demands and exhaustion; items with high loadings on Factor 3 corresponded to worry and anxiety subscales of the TICS and PSQ. Thus, the three factors were labeled as Stress due to *Lack of Resources*, *Burden*, and *Worry*, respectively.

#### Emotion Regulation

We used the German version of the *Cognitive Emotion Regulation Questionnaire* (CERQ; Garnefski and Spinnhoven, 2006, German adaptation by Loch et al., 2011) to assess emotion regulation. Only the subscales that correspond to positive emotion regulation strategies (i.e., “Acceptance,” “Positive Refocusing,” “Refocus on Planning,” “Putting into Perspective,” and “Positive Reappraisal”) (Garnefski et al., 2005) were used for analysis. Cronbach’s alpha for the five subscales was 0.73. Maximum likelihood factor analysis with promax oblique rotation was applied on these five subscales, with one factor extracted based on the results of Horn’s parallel analysis (Glorfeld, 1995). A composite score was derived using the regression scores from the factor analysis, which we labeled as *Positive Emotion Regulation*.

#### Social Support

It has been suggested that older adults’ perception of available social support is a stronger predictor of well-being than the actual support they receive (Auslander and Litwin, 1991; Newsom and Schulz, 1996). To have a measure for both practical and perceived support, we used two subscales of *Fragebogen zur Sozialen Unterstützung* (*F-SozU*; Fydrich et al., 2009) that correspond to the receipt of *Practical Social Support* (PSS) as well as the personal *Satisfaction with Social Support* (SSS) received. Cronbach’s alpha for these subscales were 0.82 and 0.65, respectively.

#### Future Time Perspective

We used the German version of the 10-item *Future Time Perspective Scale* (FTP; Carstensen and Lang, 1996) to assess perceived limitations on time. Sample items in this scale include: “Many opportunities await me in the future” and “Most of my life (still) lies ahead of me.” Answers were given on a 7-point Likert-type scale, ranging from 1 (*strongly disagree*) to 7 (*strongly agree*). Cronbach’s alpha for this subscale was 0.87.

#### Cognition

We chose measures of cognition based on previous research relating top-down emotion regulation to executive functioning (Ochsner and Gross, 2005). We derived a score for cognitive and executive function across the domains of speed of processing, attention/vigilance, working memory, and visual learning. For this purpose we used the following tests: (1) Part A and B from the *Trail Making Test* (TMT; Reitan, 1992), the 2-back task from *Testbatterie zur Aufmerksamkeitsprüfung* (TAP; Zimmermann and Fimm, 2012) using mistakes and omissions, and the Simon-effect score (Simon and Small, 1969) for working memory; (2) The LPS 4 (subtest 4 of the German intelligence test battery *Leistungsprüfungssystem* (LPS; Horn, 1983) for fluid intelligence; (3) The two subscales of the *Regensburger Wortflüssigkeitstest* (RWT; Aschenbrenner et al., 2000) for verbal memory, and a learned sum score of *California Verbal Learning Test* (CVLT; Niemann et al., 2008). Cronbach’s alpha for these subscales was 0.63. We used *Very Simple Structure* in R in order to screen for the number of factors. The VSS complexity 1 suggested one factor solution with a fit value of 0.75, which was congruent with the number that was suggested by the Velicer Minimum Average Partial (MAP; Velicer, 1976) criterion. A composite score was derived for cognitive functioning for each participant using the regression scores from the maximum likelihood factor analysis with a promax oblique rotation.

### Stimuli and Apparatus

Face stimuli were used to measure visual attention to emotional stimuli. These stimuli consisted of high-resolution, front-view photographs of Caucasian faces that were taken from the FACES database^5^ (Ebner et al., 2010). “Happy,” “angry,” “sad,” and “neutral” facial expressions from 144 individuals were randomly selected from a pool of 171 individuals using a customized MATLAB (MathWorks, Natick, MA, United States) script. Two emotional expressions from each of these 144 individuals were again randomly selected for each participant from the pool of 576 pictures (i.e., 4 emotion categories from 144 faces) and presented in a counterbalanced fashion for age (young/middle/old), sex (female/male), emotion type (happy/angry/sad/neutral), and picture presentation position (left/right).

Stimuli were presented using Presentation Software (Neurobehavioral Systems Inc., Berkeley, CA, United States). We recorded participants’ left eye movements with a sampling rate of 1,000 Hz using an Eyelink 1,000 eye tracker system (SR Research Ltd., Ottawa, Canada). A chin-rest was used to limit head movements. The stimuli were presented on a 22-inch Ilyama CRT monitor positioned 80 cm away from the participant.

### Task Design and Procedure

Before the recording session, participants performed a 9-point eye calibration test in order to ensure an accurate mapping between the eye orientation and eye tracker measurements. Next, participants were presented with a sequence of face pairs—two pictures of the same person would appear simultaneously on the left and right side of a fixation cross—and were instructed to “view the images naturally as if at home watching television” (Isaacowitz et al., 2006). In 144 trials, participants were presented with 10 combinations of facial emotions: 48 trials, in which both faces showed the same emotion (happy–happy, sad–sad, angry–angry, neutral–neutral) and 96 trials, in which the emotion differed between the left and the right face (happy–sad, happy–angry, happy–neutral, neutral–angry, neutral–sad, sad–angry). The latter were counterbalanced for positive–negative, positive–neutral, neutral–negative, and negative–negative contrasts. Each picture (pairing of an individual face and a specific emotion) appeared only once for each participant. Trials, in which both faces showed the same emotion (e.g., happy–happy), were used as filler trials to mask the aim of the study. Each pair was presented for 3,000 ms with a 1,500 ms fixation cross between trials. Participants were tested in a quiet room and left alone during the eye-tracking session.

### Data Analysis

Eye tracker files were processed using customized MATLAB (MathWorks, Natick, MA, United States) scripts, and statistical analyses on the fixations were carried out using the R Software (Version 3.1, R Development Core Team, 2008). As a first step, trials with the same emotional expression on both sides were discarded. Since the experimental stimuli were presented in pairs, for the remaining 96 trials of happy–angry, happy–sad, happy–neutral, neutral–sad, neutral–angry, sad–angry face pairs, *Fixation Ratio Scores* (FRS; Isaacowitz et al., 2006) were calculated to assess relative looking patterns on both side of these pairs. We used durations rather than number of fixations, given that this is generally considered to be a better proxy for capturing attentional biases (Allard and Isaacowitz, 2008).

Percent of total fixation duration on each side of face pairs for each contrast were quantified for each participant relative to the total duration of fixations on both pairs for the entire 3,000 ms trial duration, and separately for the first (0–1,500 ms) and second half (1,500–3,000 ms) of the trial duration time. A fixation ratio over 50% would indicate gaze preference toward the more positive face.

We carried out two ANOVA analyses to investigate overall age differences: (1) For the entire 3,000 ms trial durations (positivity bias) using a two-way mixed-effects ANOVA with Age Group (younger/older adults) as a between-subject variable, and five emotion contrasts (happy–angry, happy–sad, happy–neutral, neutral–sad, and neutral–angry) as within-subject variables, and percent of total fixation duration on happy (in happy vs. angry/sad/neutral), and neutral (in neutral vs. angry/sad pairings) faces in these contrasts as the dependent variable,^6^ (2) by adding time course to the previous analysis: with a 2 (Age Group) × 2 (Time Course: Early/Late Trial Duration) × 5 (Emotion Contrasts) mixed effects ANOVA, in order to examine whether younger and older adults’ gaze preferences for the more positive face were different in late (last 1,500 ms) compared to the early trial periods (first 1,500 ms) (*positivity shifts*).^7^ A potential Age Group × Time Course interaction would implicate that the time course of positivity differs between younger and older adults.

Next, we quantified an index for positivity shifts for each participant to conduct further analyses. We subtracted the percent of total fixation durations on the “more positive” side (i.e., to happy faces paired with negative and neutral faces, and to neutral faces paired with angry or sad faces) of pairs of the first half (first 1,500 ms) from the second half (last 1,500 ms) of the entire trial duration (3,000 ms). We then quantified individual levels of positivity shift (*the positivity shift index*) by taking the average of these five values, in order to perform regression analyses to examine our hypotheses related to the role of inter-individual differences.

We performed two separate analyses on positivity shifts: (1) We tested the moderator role of age on the relationship between the positivity shift and FTP. We used age as a categorical variable interacting with FTP to investigate differential effects of age group with FTP. Predictors were centered (*z*-scored), a method suggested in order to reduce multicollinearity (Cohen et al., 2003). Significant interactions were subsequently probed by simple slope analyses.

(2) Using multiple regression analysis, we examined how other socioemotional predictors alongside age are associated with positivity shifts. Age was entered in the analyses as a continuous variable interacting with the predictors to capture possible non-linear interactions between age and the predictors. All predictors were again centered before computing the interaction term. *Variance Inflation Factors* (VIF) and their tolerance indices were used to assess how much the variance of the coefficient estimate is being inflated by multicollinearity. Predictors were again centered (*z*-scored). The VIF scores for the multiple regression analyses were all below five.

We first compared two models. In the full model, the positivity shift score was regressed onto age, interacting with stress (lack of resources, burden, worry), FTP, practical social support, satisfaction with social support, positive emotion regulation, cognitive functioning, and well-being. The null model comprised only age as a continuous variable predicting positivity shifts. The reduced model comprised significant primary interactions from the initial model.

Pearson’s correlations were used to evaluate the relationships between the study variables in the two age groups separately (see Table 2). We used r-to-z transformations to compare correlations between younger and older adults. For all subsequent statistical analyses, we used a two-sided alpha level of 0.05 with 95% confidence intervals, and Cohen’s *d* or partial eta squared (η*_*p*_*^2^) for measuring the effect size.

## Results

### Age Group Differences in Gaze Preferences to Face Pairs

#### Positivity Bias

We examined overall age differences in fixation durations on happy and neutral face stimuli (in happy vs. angry/sad/neutral, and neutral vs. angry/sad contrasts) for the entire 3,000 ms trial period (*positivity bias*) using a 2 (Age Group) × 5 (Contrast Type) mixed-effects ANOVA. The main effect of age group was not significant, *F*(1, 127) = 0.45, *p* = 0.502, η*_*p*_*^2^ = 0.00, indicating no overall significant age difference between younger and older adults’ preferences to more positive stimuli when the entire 3 s trial period was considered.

#### Positivity Shifts

**FIGURE 1:**
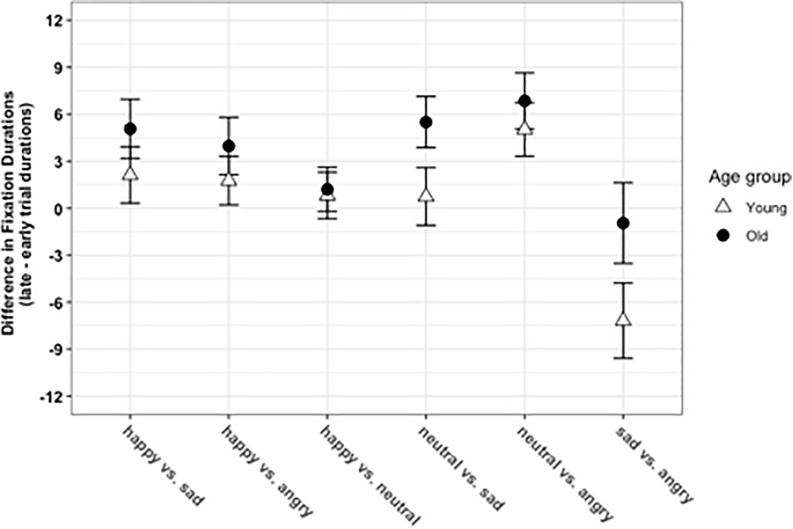
Fixation shifts of younger and older adults on face pairs as measured by the eye tracker. Error bars represent mean shifts; 0 represents zero shifts to either side of a pair, values over 0 represent more shifts to the first emotional expression on each pair on the *x*-axis.

Fixation durations on the more positive side of the five contrasts in late compared to the early trial periods examined by 2 × 2 × 5 mixed-effects ANOVA revealed a significant interaction between Age Group × Time Course, *F*(1, 127) = 4.68, *p* = 0.032, η*_*p*_*^2^ = 0.03, and between Age Group × Contrast Type, *F*(4, 1,018) = 5.65, *p* < 0.001, η*_*p*_*^2^ = 0.02 (see Figure 1). *Post-hoc* pairwise Tukey *t*-tests showed that the fixation durations on more positive stimuli in late (*M* = 56.7, *SD* = 1.64) compared to early trials (*M* = 52, *SD* = 1.19) were significantly different in older adults *t*(127) = −4.11, *p* < 0.001, *d* = 0.58, but not in younger adults (early *M* = 51.7, *SD* = 0.72, late *M* = 53.5, *SD* = 1.22), *t*(127) = −1.41, *p* = 0.493, *d* = 0.17, indicating that older adults’ fixations on more positive faces increased in the late trial period. Although we were mainly interested in overall age-related differences in positivity in early and late trials, we also examined the interaction between Age-group × Contrast, however, we found no age-group differences for any relevant contrast for the two age groups. The Age Group × Time Course × Contrast Type was not significant (*p* = 0.718).

#### Fixation Durations on Sad vs. Angry Faces in Early/Late Trial Periods

Age differences in gaze preferences to sad vs. angry face stimuli in late compared to early trial durations were examined using a two-way mixed-design ANOVA, with Age Group (younger/older adults) as the between-subject factor, Time Course (early/late) as within-subject factor, and percent of total fixation duration on sad faces as the dependent variable. This analysis revealed a significant main effect of Time Course, *F*(1, 252) = 6.98, *p* = 0.008, η*_*p*_*^2^ = 0.02. Age Group, *F*(1, 252) = 2.88, *p* = 0.090, η*_*p*_*^2^ = 0.01, and the interaction between Age Group × Time Course, *F*(1, 252) = 2.98, *p* = 0.085, η*_*p*_*^2^ = 0.01, were not statistically significant.

#### Fixation Durations on Upper or Lower Parts of Faces

We tested whether younger and older adults showed differences in attending to upper (i.e., toward the eye region of faces) or lower part (i.e., more mouth region of the face) of facial stimuli. Younger adults had overall longer durations of fixations on the upper part of faces than older adults, *t*(92) = 2.77, *p* = 0.006. A two-way mixed-design ANOVA, with Age Group (younger/older adults) as the between-subject factor, four emotion categories (happy/sad/angry/neutral) as within-subject factor, and percent of total fixation duration on the upper part of these four emotional expressions as the dependent variable showed that the effect of the age group was significant, [*F*(1, 3) = 37.73, *p* < 0.001], indicating that older adults’ fixations on lower part of faces were significantly longer across happy, sad, angry, and neutral faces. Linear regression analysis where positivity shifts were regressed onto percentage of fixation durations on upper part of faces in interaction with the age group was not statistically significant [*F*(2, 126) = 1.67, *p* = 0.192], indicating that fixation durations on upper or lower parts of faces were not significantly associated with positivity shifts in neither group.

### Moderating Role of Age for the Relationship Between Positivity Shift and FTP

**FIGURE 2:**
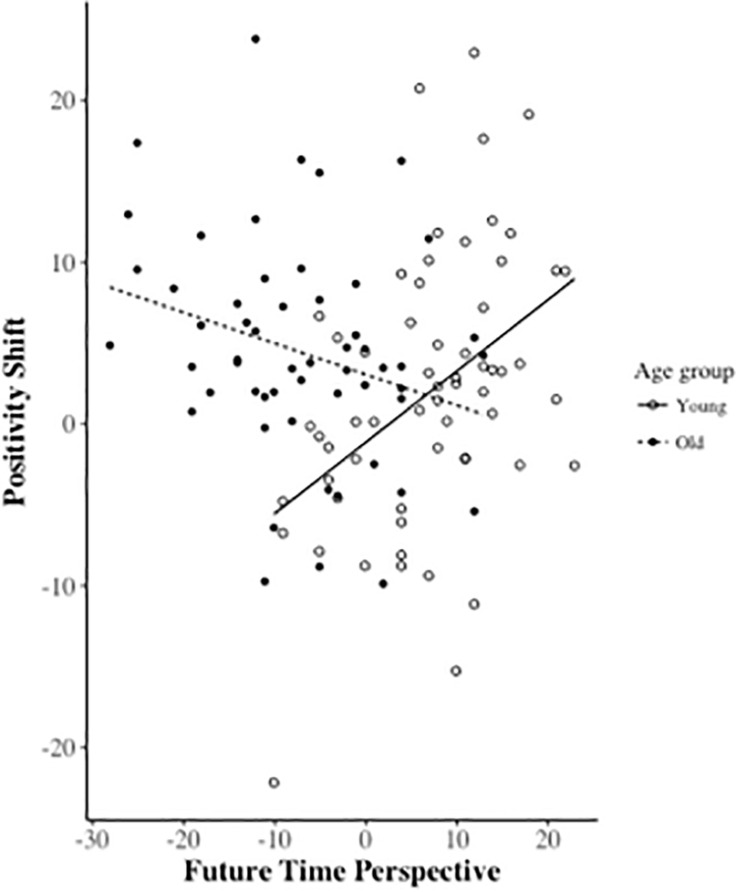
Age × FTP interaction for the positivity shift. Positivity shift and FTP are mean centered by age group. Lines represent lines of best fit from the regression analysis.

We examined whether the relationship between positivity shifts and FTP is moderated by age. Positivity shift scores were regressed onto FTP, Age Group, and the interaction between FTP and Age Group. Age Group, (*b* = 4.17, *SEb* = 1.73, β = 0.26, *p* = 0.017), and FTP, (*b* = 0.44, *SEb* = 0.44, β = 0.64, *p* < 0.001) showed significant main effects, qualified by a significant interaction between FTP × Age Group (*b* = -0.63, *SEb* = 0.15, β = -0.61, *p* < 0.001), indicating that FTP’s association with positivity shifts was positive in younger (*b* = 0.44, *SEb* = 0.11, *p* < 0.001), and negative in older adults (*b* = -0.19, *SEb* = 0.10, *p* = 0.061) (see Figure 2).

To test whether FTP might have different meanings across age, we additionally examined correlations between our predictors (see Table 2). Comparing correlations using r-to-z transformation, we assessed whether the correlation between FTP and socioemotional functioning variables (e.g., well-being, and worry) differ in magnitude in the younger and in the older adults. FTP was positively associated with well-being levels in the younger, and negatively in the older adults (Younger: *r* = 0.47, *p* < 0.001, Older: *r* = 0.16, *p* = 0.19, r-to-z value: *z* = 1.95, *p* = 0.02). FTP’s association to worry also differed between the younger and older adults (Younger: *r* = -0.31, *p* < 0.01, Older: *r* = 0.11, *p* = 0.41, r-to-z value: *z* = −2.36, *p* < 0.001).

### The Role of Inter-individual Differences in Positivity Shifts

To examine whether predictors related to socioemotional functioning that we measured alongside age are associated with positivity shifts, multiple regression analyses were performed. To test the significance of the predictors as a whole, we compared the fit of the full model (where the positivity shift score was regressed onto age, lack of resources, burden, worry, FTP, practical social support, satisfaction with social support, positive emotion regulation, cognitive functioning, and well-being) with that of the null model comprising only age. Age was entered as a continuous variable in interaction with the other predictors to examine whether within group differences in predictors differentially relate to positivity shifts across age. Overall, the comparison of the full model with all the predictors to the null model that comprised only age was significant [*F*(19, 93) = 1.97, *p* = 0.019]. The full model revealed a significant regression equation [*F*(19, 93) = 2.09, *R*^2^ = 0.29, *p* = 0.010]. Age and FTP showed a significant interaction (*b* = -0.01, *SEb* = 0.00, *p* < 0.001). Non-significant interactions from the full model were removed to form the reduced model which comprised Age and FTP interaction, and the remaining predictors. Along with a significant Age and FTP interaction, worry had a significant effect (*b* = −1.99, *SEb* = 0.92, *p* = 0.034), indicating a negative association with positivity shifts across age (for full and reduced models see Table 3).

**TABLE 3:** Summary of the initial multiple regression analysis with all predictors, and the reduced model with significant interactions from the initial model.

Predictor	*b*	*b* 95% CI	*sr*^2^	*sr*^2^ 95% CI	Fit
(Intercept)	0.69	[−1.95, 3.33]			
Age	1.16	[−1.80, 4.13]	0.00	[−0.02, 0.03]	
Lack of resources	–1.65	[−3.91, 0.60]	0.02	[−0.02, 0.05]	
Burden	–1.20	[−3.05, 0.64]	0.01	[−0.02, 0.05]	
Worry	−2.03*	[−3.95, -0.11]	0.03	[−0.02, 0.09]	
Future time perspective	2.23*	[0.07, 4.39]	0.03	[−0.02, 0.09]	
Satisfaction with social support	–0.80	[−2.59, 0.99]	0.01	[−0.02, 0.03]	
Practical social support	–0.18	[−2.18, 1.83]	0.00	[−0.00, 0.00]	
Positive emotion regulation	–0.37	[−1.97, 1.23]	0.00	[−0.01, 0.01]	
Cognition	–0.44	[−2.97, 2.08]	0.00	[−0.01, 0.01]	
Well-being	–0.28	[−2.25, 1.68]	0.00	[−0.01, 0.01]	
Age × Lack of resources	2.20	[−0.06, 4.45]	0.03	[−0.02, 0.08]	
Age × Burden	1.16	[−0.61, 2.93]	0.01	[−0.02, 0.05]	
Age × Worry	0.73	[−1.20, 2.66]	0.00	[−0.02, 0.02]	
Age × FTP	−3.53**	[−5.40, -1.66]	0.11	[0.01, 0.20]	
Age × Satisfaction with social support	1.40	[−0.44, 3.25]	0.02	[−0.02, 0.06]	
Age × Practical social support	1.69	[−0.43, 3.82]	0.02	[−0.02, 0.06]	
Age × Positive emotion regulation	0.53	[−1.11, 2.18]	0.00	[−0.01, 0.02]	
Age × Cognition	–0.64	[−3.09, 1.81]	0.00	[−0.01, 0.02]	
Age × Well-being	–0.13	[−2.00, 1.73]	0.00	[−0.00, 0.00]	
					*R*^2^ = 0.29* 95%CI [0.02, 0.31]
(Intercept)	1.27	[−0.51, 3.06]			
Age	0.94	[−1.95, 3.83]	0.00	[−0.02, 0.02]	
Future time perspective	1.53	[−0.52, 3.58]	0.02	[−0.03, 0.06]	
Lack of resources	–0.99	[−3.14, 1.16]	0.01	[−0.02, 0.03]	
Burden	–0.75	[−2.51, 1.01]	0.01	[−0.02, 0.03]	
Worry	−1.99*	[−3.84, -0.15]	0.04	[−0.03, 0.10]	
Satisfaction with social support	0.07	[−1.62, 1.76]	0.00	[−0.00, 0.00]	
Practical social support	–0.57	[−2.46, 1.31]	0.00	[−0.01, 0.02]	
Positive emotion regulation	–0.10	[−1.68, 1.49]	0.00	[−0.00, 0.00]	
Cognition	–0.53	[−2.88, 1.81]	0.00	[−0.01, 0.01]	
Well-being	–0.22	[−2.07, 1.62]	0.00	[−0.01, 0.01]	
Age × FTP	−2.98**	[−4.70, -1.27]	0.09	[−0.00, 0.19]	
					*R*^2^ = 0.21** 95%CI [0.02, 0.27]

A significant b-weight indicates the semi-partial correlation is also significant. b represents unstandardized regression weights. sr^2^ represents the semi-partial correlation squared. *p < 0.05. **p < 0.01.

## Discussion

We investigated younger and older adults’ gaze preferences on different pairings of angry, sad, happy, and neutral face stimuli, and how inter-individual factors related to socioemotional functioning, most relevant FTP, might be associated with greater attention to the more positive faces. While we did not observe any significant age-group difference in overall gaze preferences to the face pairs when the entire trial duration was considered, older adults showed greater attentional shifts to more positive stimuli in later stages of processing (i.e., *positivity shifts*). As expected, FTP was more limited in the elderly group. Interestingly, however, age moderated the relationship between positivity shifts and FTP, such that the relationship between the FTP and positivity shifts was positive in the younger, and negative in the older adults. While positivity shifts in younger adults were modulated by inter-individual differences related to socioemotional functioning, this was not the case in older adults. Similarly, the relationship between FTP and other socioemotional factors also differed across age.

### Age Similarities and Differences in Gaze Preferences to Emotional Stimuli

Our results support the existence of a positivity effect, but suggest that an age-dependent group difference in attention toward “positive information” needs to be qualified by additional parameters. We found that the age-dependent group effect only emerged when the temporal dynamics of gaze patterns were examined. Specifically, although both groups showed strikingly similar gaze patterns on face pairs and more attention to the more positive face during later stages of processing (see Figure 1), gaze preferences for the more positive face in late compared to early trial durations were significantly stronger in the older adults. A delayed occurrence of positivity seems consistent with an emotion regulatory mechanism which takes into account the first (random sequence) observations and subsequently guides attention toward the more positive image. Thus, this finding might support the argument that the positivity effect might reflect motivated cognition in older adults to a greater extent than it does in younger adults, and is congruent with previous studies which also showed a delayed onset of positivity in older adults (Isaacowitz et al., 2009; Lee and Knight, 2009).

Furthermore, results from the ANOVA analysis gave no indication that age differences in positivity shifts differentially emerged for a particular contrast (for happy vs. sad, happy vs. angry, happy vs. neutral, neutral vs. angry, and neutral vs. sad contrasts). Based on previous reports regarding the differential processing of particularly negative emotions such as sadness and anger across age (Blanchard-Fields and Coats, 2008; Streubel and Kunzmann, 2011; Kunzmann et al., 2014), we conducted an exploratory analysis to test whether younger and older adults show a differential preference for sad or angry faces (in sad vs. angry contrast) in late compared to early trial durations, but failed to observe a significant Age × Emotion interaction. Both groups fixated longer on the angry face in late compared to early trial durations (see also Figure 1).

We also examined whether there are age-related differences in focusing on different regions of faces, and whether these relate to positivity shifts. In line with previous findings, younger adults spent more time looking at the eye regions, whereas older adults on mouth regions, independent of the emotional expression displayed. This has been suggested to be a compensatory mechanism for hearing loss that often accompany increasing age, through older adults’ increased reliance on visible speech (Thompson, 1995). We had hypothesized that increased positivity in gaze preferences to more positive faces might be related to this age-related differentiation in focusing on different regions of faces, however, our results did not support this link. Eyes provide social cues, so it might be the case that older adults miss on important aspects of communication due to this trade-off. However, this did not seem to be related to greater shifts to positive faces in older age, possibly since older adults fixated longer at the mouth region of the faces independent of the emotion displayed.

Taken together, our results show that younger and older adults’ positivity preferences did not significantly differ according to the stimulus type (i.e., groups showed more similarities than differences in their gaze preferences to face pairs), and for each contrast, both groups on average showed a shift toward the more positive face (when comparing late vs. early trial durations, see Figure 1). However, the overall shifts to more positive faces were significantly higher for older compared to younger adults, supporting SST’s notion that a motivational shift to more positive, emotionally gratifying information increases with age.

### The Role of FTP in Positivity and Socioemotional Functioning in Younger and in Older Adults

Previous reports have emphasized the role of limited FTP in the positivity effect using the theoretical framework of SST. Despite its presumably important role, a few studies that have used a questionnaire-based FTP did not observe any significant links to behavioral assessments of positivity (e.g., Demeyer and De Raedt, 2013; Bohn et al., 2016). Rather than manipulating participants’ perceived future time by asking them to imagine a limited or an open-ended FTP (which was the method mostly used by previous studies), we investigated whether how one perceives future time would moderate the relationship between positivity shifts and age. We reasoned that between-person differences in FTP would differentially be related to positivity across age groups, based on the assumption that perceiving one’s remaining life as limited (as operationalized by the FTP questionnaire) might have a different meaning, and therefore different behavioral outcomes for younger and older adults. One important aim of our study was to investigate this hypothesis.

Regarding the age-related differences, we confirmed that FTP was more limited in the older group, who also showed stronger gaze preferences for the more positive face. However, we found that the interaction between FTP and the age group in predicting the positivity shift was significant (see Figure 2). In line with our prediction, FTP was more negatively associated with positivity shifts in the older group, while a more positive relationship was observed in the younger adults. For the older group, increased preference for positive stimuli especially in the later stages of processing (e.g., more controlled as opposed to automatic, reflexive processes) being driven by a limited FTP seems consistent with the tenet of the SST that age-related positivity is related to a change in FTP. However, this did not seem to apply to the younger people in our study. This is similar to Kellough and Knight’s (2012) finding, where the authors experimentally manipulated participants’ time perspective, and found that while imagining a more expanded life span decreased positivity in older adults’ perception of emotions, imagining a limited FTP did not have an effect on younger adults’ perception of emotions. Although the FTP has been measured differently in these studies, these similar findings regarding the moderator role of age suggest that positivity in information processing depend on both age and FTP.

The different outcomes of FTP on positivity as a function of age might be related to a different meaning that a limited FTP might have for the two age groups. In line with this, we observed that FTP’s associations with worry and well-being levels showed significant differences between the age-groups, such that the negative associations of FTP to worry and well-being were more significant in the younger adults.

While FTP is claimed to be the driving force behind the positivity effect and higher levels of well-being in the elderly, Grühn et al. (2016) showed FTP to be inversely related to socioemotional functioning independent of age. In our study, an open-ended FTP indicated a more adaptive socioemotional profile in younger adults, given its highly significant association with well-being, where in older adults no such link was observed. The FTP measure used in our study, as conceptualized by Lang and Carstensen (2002), captures whether an individual perceives the future in positive or negative light. One of the questions included in the FTP scale is to what extent individuals perceive their future to be full of opportunities. One would expect an older person’s answer to this question to be less affirmative than a younger person’s. For a younger person with a more limited or constrained outlook into the future, the negative consequences for their current well-being can be deemed expected. On the other hand, for an older individual, not perceiving the future to be full of opportunities would most probably have a lessened impact on their current well-being. In other words, it might be possible that the different ends of the scale implicate different things for younger and older adults, and that the relationship between the instrument (the FTP questionnaire) to the underlying construct is not linear. While the SST treats FTP as a unidimensional construct, it has been suggested that FTP is a two-factor or multidimensional construct (Cate and John, 2007). While this is not elaborated in the theory, some authors suggest that only specific facets of FTP might indicate positive socioemotional functioning. Our findings, along with the mixed findings observed about the links between FTP and socioemotional functioning, show that the relationship is nuanced. While more data are needed to draw firmer conclusions, based on our findings—particularly the relationship between FTP and well-being—we suggest that FTP (as assessed by the FTP measure) might reflect different socioemotional profiles depending on the phase of life. Future studies should consider the potential qualifier role of age in their assessments of FTP along with positivity in information processing or socioemotional functioning.

### The Role of Inter-individual Differences in Positivity

Based on our assumption that inter-individual differences in abilities, resources, or emotional states might contribute to an individual’s top-down motivation for positive stimuli, we conducted exploratory analyses to look at the role of stress, emotion regulation, social support, well-being, cognitive functioning, and FTP, examining the effect of these alongside age in positivity shifts. Interestingly, we found that inter-individual differences in socioemotional factors were more significantly related to positivity shifts than age by itself, supporting the notion that age alone is not a sufficient causal factor for psychological processes (e.g., Baltes and Lang, 1997; Baltes et al., 2006), such as the positivity effect. These analyses also confirmed the interaction between age and FTP.

Some of the relationships that we hypothesized were not supported (i.e., the role of well-being, social support, and emotion regulation in positivity shifts). Although the positivity effect is claimed to be a top-down, motivationally driven process that relies on cognitive resources, our study failed to demonstrate a significant role for cognitive functioning for top-down, motivational shifts to more positive stimuli. This is consistent with a recent study which examined the association of positivity in memory and attention with performance on cognitive tasks, and found limited evidence (Barber et al., 2020). Unlike studies that associated cognitive functioning with positivity effects, studies that used manipulations of cognitive resources have shown different results. For instance, a previous study that examined the role of emotionally valanced distractors on the encoding of negative stimuli found no interference effects for positive distractors in memory for negative targets, supporting the role of autonomic rather than effortful processing of positive distractors (Ziaei et al., 2015). Likewise, Mather and Carstensen (2003) found that compared to younger adults, older adults avoided emotionally negative faces during a cognitive task, supporting the claim that positivity relies on cognitive resources.

Fox and Knight (2005) examined the hypothesis whether trait and state anxiety mediated the relationship between positivity and cognition, and showed although there was a tendency for older adults without anxiety to avoid attending to negative stimuli during a cognitive task, older adults who were induced into anxious moods showed selective attention to threat similar with younger adults. This suggests an important role for anxiety in positivity in emotion processing. Consistent with this, among the stress measures that we examined (i.e., *stress due to lack of resources*, *worry*, and *burden*), worry was significantly related to positivity shifts, in that higher levels of worry were associated with fewer shifts to more positive faces across age.

Worry is characterized by “a chain of thoughts and images, negatively affect-laden, and relatively uncontrollable; it represents an attempt to engage in mental problem-solving on an issue whose outcome is uncertain but contains the possibility of one or more negative outcomes; consequently, worry relates closely to the fear process” (Borkovec et al., 1983, p. 10). For these reasons, worry is a common feature of most anxiety disorders (Beck and Clark, 1997). Significant attentional biases to negative or threatening stimuli have been reported in individuals high in trait anxiety (Quigley et al., 2012), and in participants who were induced into an anxious mood (Goodwin et al., 2017). Our finding that higher levels of worry are associated with fewer shifts to positive faces is congruent with these studies, however, this contrasts with claims that younger and older adults use positive gaze for different reasons (such that older adults use positive gaze in negative mood states, whereas younger adults show more mood congruent looking patterns) (Isaacowitz et al., 2008; Isaacowitz and Noh, 2011).

Indeed, increased positivity in older as compared to younger adults might as well be due to decreased levels of worry in older age, a very significant age-group difference that we also observed in our data (see Table 1), similar with past research (Hunt et al., 2003). For instance, worries about finances and social events are more frequent in younger than in older adults (Powers et al., 1992). It has been noted that psychological distress and mental health problems have become more widespread among the younger populations over the last twenty years (Sweeting et al., 2010; Mojtabai and Jorm, 2015), which have been linked to changes in national labor and market trends (Lager and Bremberg, 2009). Employment and the labor markets during the 1970s were “secure,” but they have since become increasingly “polarized and precarious” (Dannefer and Huang, 2017). For instance, the crises of 1980s brought about an increase in the number of suicides in Spain, which coincided with greater increases in unemployment (McKee and Stuckler, 2001). Thus, the increase in precariousness might be the reason for increased levels of anxiety among the young populations. If worry in younger populations is increasing due to social and economic changes, this change might hypothetically also be reflected in the positivity levels of younger adults (such as in emotion processing, or in affect domains), and to the age-related difference observed in these groups. The reliance of the positivity effect on worry could explain why the positivity effect, which has initially been identified and conceptualized in 2005 by Mather and Carstensen, might be influenced by cohort effects. This would also explain the inconsistencies found in previous research. For instance, age-related differences to positive over negative stimuli observed in US Americans were not observed in Hong Kong Chinese (Fung et al., 2008). Cohort effects have also been established on well-being, in that the well-being of cohorts in the US that lived through the economic challenges of the early twentieth century was lower than those born during more prosperous times (Sutin et al., 2013). Possibly also related to this, the strongest negative correlate of well-being in our data was lack of resources, which depicted satisfaction with one’s job and social conditions.

From this perspective, a differential relationship of FTP to positivity shifts in younger and older adults and the stronger link between perceived limited FTP and worry in younger adults would make sense. This is possibly because FTP comprises ideas such as “many opportunities lie ahead of me in the future.” Younger adults who are pessimistic about their future and who think the future does not bear many opportunities for them might be more prompted to focus on the negative information.

One is more prompted to focus on the negative when one feels unsafe or threatened. When one feels more secure, it might be easier to afford to pay attention to the positive in the environment. There is little reason as to why this should be different in younger and in older adults. Rather than focusing on age-group differences, studies should try to identify why these differences emerge between younger and older groups, and include inter-individual assessments related to socioemotional functioning. Taken together, our study extends the prior literature by showing that along with the interaction between Age and FTP, lower levels of worry are important factors for increased positivity in emotion processing in general, pointing to the necessity of studying inter-individual differences in psychological constructs in positivity effect research.

### Limitations, Caveats, and Future Directions

An important limitation of our study lies in its cross-sectional nature and its heavy reliance on self-report data. Drawing on the findings that the positivity effect mostly emerges when participants are free from experimental constraints (Reed et al., 2014), we chose a relatively simple task, in which the participants passively viewed pairs of face stimuli. Since this kind of task does not implicate any emotional response on the part of the participants, they cannot be claimed to test emotion regulation (Isaacowitz and Blanchard-Fields, 2012). Since we did not manipulate gaze or other measures we used (i.e., well-being, emotion regulation, FTP, etc.), but rather examined associations between behavioral assessments of positivity and self-report data in light of our hypotheses, we cannot draw conclusions about causal pathways underlying the positivity effect and associated outcomes for well-being. Studying age-related changes in emotion processing with ambulatory assessments and/or in longitudinal designs would be more desirable to establish such links.

An important focus of our study was to investigate the theoretical grounding of the positivity effect. We therefore investigated the role, and indirectly the meaning of FTP, as it has been treated as the most relevant construct to explain the age differences in emotion processing. Since previous reports have claimed increased positivity—suggested to be driven by a limited FTP—to have a potential role for the well-being of older adults (Reed and Carstensen, 2012), it would be interesting to test the role of well-being levels in the relationship between positivity and FTP, and whether this relationship might differ across the age groups. While our study did not have enough statistical power for this three-way interaction, we provide data on this exploratory analysis (detailed results are reported in the Supplementary Material). We observed that the negative relationship between FTP and positivity indicated older adults with lower well-being levels. Since this analysis did not have sufficient statistical power, its results are speculative at this point. Nevertheless, we regard this worthwhile to be pursued further (in studies with adequate power). Our data will be publicly available for future meta-analyses.

Another important thing to add is that the older adults in the current study, in contrast to previous research (Mroczek and Kolarz, 1998), did not report higher well-being as compared to younger adults. Surprisingly also, younger adults in our study reported using more positive emotion regulation than older adults. It might be worth noting that most of the older adults who took part in this study have lived in the former German Democratic Republic (GDR). With the fall of the Berlin Wall, people who had lived in the GDR suddenly experienced a great amount of instability and change. The experience of stability and continuity is essential to an individual’s well-being (Westerhof and Keyes, 2006). Thus, one reason that older adults did not report higher life satisfaction than younger adults might be related to this, given that the age-related differences in positive affect are suggested to be small, and hard to disentangle from cohort effects (Kunzmann et al., 2000). Future studies might consider including measurements of important life events (such as important personal or health-related changes), measures related worry, anxiety, job and social security, and whether positivity in older adults is still maintained in face of a potential health threat (such as a pandemic like Covid-19 which poses a stronger risk for elderly people), which could provide new insights into the positivity effect and its relationship to socioemotional functioning.

## Conclusion

The positivity effect, i.e., increased attention and memory for positive information in older as compared to younger people is thought to play a major role in emotional well-being in older age. Within the framework of SST, the positivity effect is suggested to be driven by a limited FTP. However, although FTP has been the most relevant construct in explaining the positivity effect, questions regarding its relationship to a top-down, motivationally driven positivity in emotion processing, and its meaning across age groups were still open. Our findings confirmed the presence of increased positivity in the older adults, who more often shifted their attention to more positive stimuli in later stages of processing (i.e., positivity shifts). We found that age moderated the association between positivity shifts and FTP, such that the positivity shifts were associated with more limited FTPs in older, and more open FTPs in younger adults, most likely stemming from a different meaning of FTP across the age-groups. Moreover, we show that neither age, nor the associated FTP *per se* were sufficient in explaining positivity shifts, and inter-individual differences in levels of worry also played a significant role. Our study is the first one to demonstrate the moderator role of age in the relationship between top-down positivity in emotion processing and FTP, with its underlying dynamics related to socioemotional functioning. Our findings also demonstrate the importance of studying inter-individual differences in socioemotional functioning rather than solely focusing on age-related differences in studies of the positivity effect.

## Data Availability Statement

The datasets generated for this study can be found in the online repositories. The names of the repository/repositories and accession number(s) can be found below: Parts of our data (all measures used in our study except the eye-tracker data) are publicly available and can be accessed via Gesellschaft für wissenschaftliche Datenverarbeitung mbH Göttingen (GWDG) (https://www.gwdg.de/). Raw and preprocessed data at this location is accessible through web browser (https://ftp.gwdg.de/pub/misc/MPI-Leipzig_Mind-Brain-Body-LEMON/) and a fast FTP connection (ftp://ftp.gwdg.de/pub/misc/MPI-Leipzig_Mind-Brain-Body-LEMON/). In the case the location of the data changes in the future, the location of the dataset can be resolved with PID 21.11101/0000-0007-C379-5 (e.g., http://hdl.handle.net/21.11101/0000-0007-C379-5).

## Ethics Statement

The studies involving human participants were reviewed and approved by the ethics committee of the Medical faculty of the University of Leipzig (reference number 154/13-ff). The patients/participants provided their written informed consent to participate in this study.

## Author Contributions

ME, AV, AB, JRo, DK, JRe, AR, LS, and MU contributed to the overall planning and data acquisition. ME, AV, and EV contributed to the design of the positivity and eye tracker task. ME with the assistance of AV, MG, and EV performed writing of the manuscript. ME with the assistance of TN, EV, MG, and AV performed the data analysis. All authors contributed to the article and approved the submitted version.

## Conflict of Interest

The authors declare that the research was conducted in the absence of any commercial or financial relationships that could be construed as a potential conflict of interest.

## Footnotes

1 In their meta-analysis, Murphy and Isaacowitz (2008) state that the terms such as *positivity bias*, *positivity effect*, and *negativity effect* have been used liberally throughout the literature to implicate biases for positive over negative, or for positive and negative over neutral stimuli. We define *positivity bias* in our study as greater preference for more positive stimuli during the entire trial periods. Time course differences in *positivity bias*—particularly a more pronounced positivity bias in late compared to early trial durations—would be identified as the *“positivity shift.”* According to the proponents of the SST (see Reed and Carstensen, 2012), it is this delayed positivity which marks the age differences in *the positivity effect*.

2 We had no a priori hypothesis regarding this contrast (sad vs. angry); rather we used this contrast as we used all pairings of happy/neutral/sad/angry faces to counterbalance the emotional stimuli in our study. This analysis was implemented *post-hoc* after finding out that previous studies have indicated differences with respect to sadness and anger in younger and in older adults (e.g., Blanchard-Fields and Coats, 2008; Kunzmann et al., 2014).

3 Some files from the eye tracker had long missing data intervals. The eye tracker camera could not reliably detect the pupil in participants wearing glasses (especially anti-reflective types) or hard lenses, or when the participant changed posture, making the files unusable for analysis. Some files were corrupt, such that the original raw files from the eye tracker could not be opened, therefore, they could not be used for further processing.

4 Factor analyses in our study were mainly used for data reduction purposes. For this factor analyses, the results were confirmed by the parallel analysis approach (Horn’s Test of Principal Components/Factors) as well as the Very Simple Structure approach in R (Dinno, 2012). The Kaiser-Meyer-Olkin (KMO) test values indicating the sampling adequacy for factor analysis for emotion regulation, stress, and for cognitive functioning scales were 0.70, 0.84, and 0.80, respectively.

5 The arousal levels of the faces have not been controlled for in this study. After the eye tracker study, participants were asked to rate 48 novel face stimuli from the FACES database. They were instructed to rate the intensity level of angry, sad, happy, and neutral faces according to how happy, angry, and sad the faces looked on a Likert-type scale from 0 (*not at all*) to 4 (*very much*). The faces were again counterbalanced for age group (young/middle-aged/old faces), sex (male/female), and emotional expression (sad/angry/neutral/happy). This task was carried out to investigate emotional perception and is not directly linked to the current study. The results will be reported elsewhere. We do not report these results here—as a manipulation check—since the stimuli in these two tasks were not the same. However, since the younger and older participants rated the same 48 faces, it might be relevant to report that we found that younger and older adults did not show differences in the overall accuracy of labeling the emotions.

6 We included participant sex as a within-subject covariate in our preliminary mixed-effects ANOVA analyses (both for positivity bias and positivity shift) to see whether it significantly relates to positivity preferences. We collapsed the data across participant sex when no main effect of sex or any interactions between sex and other variables emerged.

7 The same analysis was carried out on late vs. early trial durations for the sad vs. angry contrast.

## References

[B1] Allard E. S. Isaacowitz D. M. (2008). Are preferences in emotional processing affected by distraction? Examining the age-related positivity effect in visual fixation within a dual-task paradigm. *Neuropsychol. Dev. Cogn. Sec. B Aging Neuropsychol. Cogn.* 15 725–743. 10.1080/13825580802348562 18819026 PMC2645630

[B2] Aschenbrenner S. Tucha O. Lange K. W. (2000). *Regensburger Wortflüssigkeits-Test: RWT.* Hogrefe: Verlag für Psychologie.

[B3] Auslander G. K. Litwin H. (1991). Social networks, social support, and self-ratings of health among the elderly. *J. Aging Health* 3 493–510. 10.1177/089826439100300404

[B4] Babayan A. Erbey M. Kumral D. Reinelt J. Reiter A. M. F. Röbbig J. (2019). A mind-brain-body dataset of MRI, EEG, cognition, emotion, and peripheral physiology in young and old adults. *Sci. Data* 6:180308. 10.1038/sdata.2018.308 30747911 PMC6371893

[B5] Baltes M. M. Lang F. R. (1997). Everyday functioning and successful aging: the impact of resources. *Psychol. Aging* 12 433–443. 10.1037/0882-7974.12.3.433 9308091

[B6] Baltes P. B. Lindenberger U. Staudinger U. M. (2006). “Lifespan theory in developmental psychology,” in *Handbook of Child Psychology: Vol. 1. Theoretical Models of Human Development*, 6th Edn, ed. Lerner R. M. (Hoboken, NJ: John Wiley & Sons), 569–664. 10.1002/9780470147658.chpsy0111

[B7] Barber S. J. Lopez N. Cadambi K. Alferez S. (2020). The limited roles of cognitive capabilities and future time perspective in contributing to positivity effects. *Cognition* 200:104267. 10.1016/j.cognition.2020.104267 32229343

[B8] Beck A. T. Clark D. A. (1997). An information processing model of anxiety: automatic and strategic processes. *Behav. Res. Ther.* 35 49–58. 10.1016/s0005-7967(96)00069-1 9009043

[B9] Birditt K. S. Fingerman K. L. Almeida D. M. (2005). Age differences in exposure and reactions to interpersonal tensions: a daily diary study. *Psychol. Aging* 20 330–340. 10.1037/0882-7974.20.2.330 16029096

[B10] Blanchard-Fields F. Coats A. H. (2008). The experience of anger and sadness in everyday problems impacts age differences in emotion regulation. *Dev. Psychol.* 44 1547–1556. 10.1037/a0013915 18999321

[B11] Bohn L. Kwong S. S. Fung H. H. (2016). Time perspective and positivity effects in Alzheimer’s disease. *Psychol. Aging* 31 574-582. 10.1037/pag0000084 26974590

[B12] Borkovec T. D. Robinson E. Pruzinsky T. DePree J. A. (1983). Preliminary exploration of worry: some characteristics and processes. *Behav. Res. Ther.* 21 9–16. 10.1016/0005-7967(83)90121-3 6830571

[B13] Carstensen L. L. Isaacowitz D. M. Charles S. T. (1999). Taking time seriously. A theory of socioemotional selectivity. *Am. Psychol.* 54 165–181. 10.1037/0003-066X.54.3.165 10199217

[B14] Carstensen L. L. Lang F. R. (1996). *Future Time Perspective Scale.* Unpublished manuscript, Stanford University, Palo Alto, CA.

[B15] Carstensen L. L. Mikels J. A. (2005). At the intersection of emotion and cognition: aging and the positivity effect. *Curr. Direct. Psychol. Sci.* 14 117–121. 10.1111/j.0963-7214.2005.00348.x

[B16] Carstensen L. L. Pasupathi M. Mayr U. Nesselroade J. R. (2000). Emotional experience in everyday life across the adult life span. *J. Pers. Soc. Psychol.* 79 644–655. 10.1037/0022-3514.79.4.644 11045744

[B17] Carstensen L. L. Turan B. Scheibe S. Ram N. Ersner-Hershfield H. Samanez-Larkin G. R. (2011). Emotional experience improves with age: evidence based on over 10 years of experience sampling. *Psychol. Aging* 26 21–33. 10.1037/a0021285 20973600 PMC3332527

[B18] Cate R. A. John O. P. (2007). Testing models of the structure and development of future time perspective: maintaining a focus on opportunities in middle age. *Psychol. Aging* 22 186–201. 10.1037/0882-7974.22.1.186 17385994

[B19] Charles S. T. Carstensen L. L. (2010). Social and emotional aging. *Annu. Rev. Psychol.* 61 383–409. 10.1146/annurev.psych.093008.100448 19575618 PMC3950961

[B20] Charles S. T. Luong G. Almeida D. M. Ryff C. Sturm M. Love G. (2010). Fewer ups and downs: daily stressors mediate age differences in negative affect. *J. Gerontol. Ser. B Psychol. Sci. Soc. Sci.* 65B 279–286. 10.1093/geronb/gbq002 20123699 PMC2981451

[B21] Cohen J. Cohen P. West S. G. Aiken L. S. (2003). *Applied Multiple Regression/Correlation Analysis for the Behavioral Sciences*, 3rd Edn Mahwah, NJ: Lawrence Erlbaum Associates Publishers.

[B22] Cohen S. Kamarck T. Mermelstein R. (1983). A global measure of perceived stress. *J. Health Soc. Behav.* 24 385–396. 10.2307/2136404 6668417

[B23] Dalgard O. S. Bjork S. Tambs K. (1995). Social support, negative life events and mental health—a longitudinal study. *Br. J. Psychiatry* 166 29–34. 10.1192/bjp.166.1.29 7894872

[B24] Dannefer D. Huang W. (2017). Precarity, inequality, and the problem of agency in the study of the life course. *Innovat. Aging* 1 1–10. 10.1093/geroni/igx027 30480120 PMC6243717

[B25] Demeyer I. De Raedt R. (2013). Attentional bias for emotional information in older adults: the role of emotion and future time perspective. *PLoS One* 8:e65429. 10.1371/journal.pone.0065429 23750261 PMC3672177

[B26] Dinno A. (2012). *Paran: Horn’s test of Principal Components/Factors R Package Version 1.5.2.* Available online at: http://alexisdinno.com/Software/files/PA_for_PCA_vs_FA.pdf (accessed March 19, 2018).

[B27] Ebner N. C. Riediger M. Lindenberger U. (2010). FACES—a database of facial expressions in young, middle-aged, and older women and men: development and validation. *Behav. Res. Methods* 42 351–362. 10.3758/BRM.42.1.351 20160315

[B28] Ellenbogen M. A. Schwartzman A. E. Stewart J. Walker C. D. (2002). Stress and selective attention: the interplay of mood, cortisol levels, and emotional information processing. *Psychophysiology* 39 723–732. 10.1017/S0048577202010739 12462500

[B29] English T. (2012). *Aging, Emotion, and Health-Related Decisions. The Role of Health Status.* Berkeley: Department of Psychology, University of California.

[B30] Faul F. Erdfelder E. Buchner A. Lang A. G. (2009). Statistical power analyses using G Power 3.1: tests for correlation and regression analyses. *Behav. Res. Methods* 41 1149–1160. 10.3758/BRM.41.4.1149 19897823

[B31] First M. B. Spitzer R. L. Gibbon M. Williams J. B. W. (1996). *Structured Clinical Interview for DSM-IV Axis I Disorders, Clinician Version (SCID-CV).* Washington, DC: American Psychiatric Press, Inc.

[B32] Fox L. S. Knight B. G. (2005). The effects of anxiety on attentional processes in older adults. *Aging Mental Health* 9 585–593. 10.1080/13607860500294282 16214707

[B33] Fung H. H. Lu A. Y. Goren D. Isaacowitz D. M. Wadlinger H. A. Wilson H. R. (2008). Age-related positivity enhancement is not universal: older Chinese look away from positive stimuli. *Psychol. Aging* 23 440–446. 10.1037/0882-7974.23.2.440 18573017

[B34] Fydrich T. Sommer G. Tydecks S. Brähler E. (2009). Fragebogen zur sozialen Unterstützung (F-SozU): Normierung der Kurzform (K-14). *Zeitschrift Med. Psychol.* 18 43–48. 10.1026//0012-1924.45.4.212

[B35] Garnefski N. Baan N. Kraaij V. (2005). Psychological distress and cognitive emotion regulation strategies among farmers who fell victim to the foot-and-mouth crisis. *Pers. Individ. Differ.* 38 1317–1327. 10.1016/j.paid.2004.08.014

[B36] Garnefski N. Spinnhoven V. (2006). Cognitive emotion regulation questionnaire – development of a short 18-item version (CERQ-short). *Pers. Individ. Differ.* 41 1045–1053. 10.1016/j.paid.2006.04.010

[B37] Glorfeld L. (1995). An improvement on Horn’s parallel analysis methodology for selecting the correct number of factors to retain. *Educ. Psychol. Measurement* 55 377–393. 10.1177/0013164495055003002

[B38] Goodwin H. Yiend J. Hirsch C. R. (2017). Generalized Anxiety Disorder, worry and attention to threat: a systematic review. *Clin. Psychol. Rev.* 54 107–122. 10.1016/j.cpr.2017.03.006 28448826

[B39] Gross J. J. (1999). Emotion regulation: past, present, future. *Cogn. Emot.* 13 551–573. 10.1080/026999399379186

[B40] Grühn D. Sharifian N. Chu Q. (2016). The limits of a limited future time perspective in explaining age differences in emotional functioning. *Psychol. Aging* 31 583–593. 10.1037/pag0000060 26691300

[B41] Grühn D. Smith J. Baltes P. B. (2005). No aging bias favoring memory for positive material: evidence from a heterogeneity-homogeneity list paradigm using emotionally toned words. *Psychol. Aging* 20 579–588. 10.1037/0882-7974.20.4.579 16420133

[B42] Horn W. (1983). *Leistungsprüfungssystem.* Göttingen: Hogrefe.

[B43] Hunt S. Wisocki P. Yanko J. (2003). Worry and use of coping strategies among older and younger adults. *J. Anxiety Disord.* 17 547–560. 10.1016/s0887-6185(02)00229-3 12941365

[B44] Isaacowitz D. M. Allard E. S. Murphy N. A. Schlangel M. (2009). The time course of age-related preferences towards positive and negative stimuli. *J. Gerontol.* 64B 188–192. 10.1093/geronb/gbn036 19279221 PMC2655166

[B45] Isaacowitz D. M. Blanchard-Fields F. (2012). Linking process and outcome in the study of emotion and aging. *Perspect. Psychol. Sci.* 7 3–17. 10.1177/1745691611424750 22888369 PMC3413281

[B46] Isaacowitz D. M. Lockenhoff C. E. Lance R. D. Wright R. Sechrest L. Riedel R. (2007). Age differences in recognition of emotion in lexical stimuli and facial expressions. *Psychol. Aging* 22 147–159. 10.1037/0882-7974.22.1.147 17385991

[B47] Isaacowitz D. M. Noh S. R. (2011). Does looking at the positive mean feeling good? age and individual differences matter. *Soc. Pers. Psychol. Compass* 5 505–517. 10.1111/j.1751-9004.2011.00374.x 21837251 PMC3152299

[B48] Isaacowitz D. M. Toner K. Goren D. Wilson H. R. (2008). Looking while unhappy mood-congruent gaze in young adults, positive gaze in older adults. *Psychol. Sci.* 19 848–853. 10.1111/j.1467-9280.2008.02167.x 18947348 PMC2760922

[B49] Isaacowitz D. M. Wadlinger H. A. Goren D. Wilson H. R. (2006). Selective preference in visual fixation away from negative images in old age? An eye tracking study. *Psychol. Aging* 21 40–48. 10.1037/0882-7974.21.1.40 16594790

[B50] Jacobs I. Sim C. W. Zimmermann J. (2015). The German TEIQue-SF: factorial structure and relations to agentic and communal traits and mental health. *Pers. Individ. Differ.* 72 189–194. 10.1016/j.paid.2014.09.003

[B51] Kellough J. L. Knight B. G. (2012). Positivity effects in older adults’ perception of facial emotion: the role of future time perspective. *J. Gerontol. Ser. B Psychol. Sci. Soc. Sci.* 67 150–158. 10.1093/geronb/gbr079 21798858

[B52] Knight M. Seymour T. L. Gaunt J. T. Baker C. Nesmith K. Mather M. (2007). Aging and goal-directed emotional attention: distraction reverses emotional biases. *Emotion* 7 705–714. 10.1037/1528-3542.7.4.705 18039037

[B53] Kunzmann U. Kappes C. Wrosch C. (2014). Emotional aging: a discrete emotions perspective. *Front. Psychol.* 5:380. 10.3389/fpsyg.2014.00380 24834060 PMC4018521

[B54] Kunzmann U. Little T. D. Smith J. (2000). Is age-related stability of subjective wellbeing a paradox? Cross-sectional and longitudinal evidence from the Berlin Aging Study. *Psychol. Aging* 15 511–526. 10.1037/0882-7974.15.3.511 11014714

[B55] Lager A. C. Bremberg S. G. (2009). Association between labour market trends and trends in young people’s mental health in ten European countries 1983-2005. *BMC Public Health* 9:325. 10.1186/1471-2458-9-325 19737380 PMC2748078

[B56] Lang F. R. Carstensen L. L. (2002). Time counts: Future time perspective, goals, and social relationships. *Psychol. Aging* 17, 125–139. 10.1037//0882-7974.17.1.125 11931281

[B57] LeDoux J. E. (1995). Emotion: clues from the brain. *Annu. Rev. Psychol.* 46 209–235. 10.1146/annurev.ps.46.020195.001233 7872730

[B58] Lee L. O. Knight B. G. (2009). Attentional bias for threat in older adults: moderation of the positivity bias by trait anxiety and stimulus modality. *Psychol. Aging* 24 741–747. 10.1037/a0016409 19739931 PMC2743240

[B59] Loch N. Hiller W. Witthoeft M. (2011). The Cognitive Emotion Regulation Questionnaire (CERQ). Psychometric evaluation of a German adaptation. *Zeitschrift Klin. Psychol. Psychother.* 40 94–106. 10.1026/1616-3443/a000079

[B60] Lohani M. Isaacowitz D. M. (2014). Age differences in managing response to sadness elicitors using attentional deployment, positive reappraisal, and suppression. *Cogn. Emot.* 28 678–697. 10.1080/02699931.2013.853648 24206128 PMC3962712

[B61] Mather M. Carstensen L. L. (2003). Aging and attentional biases for emotional faces. *Psychol. Sci.* 14 409–415. 10.1111/1467-9280.01455 12930469

[B62] Mather M. Carstensen L. L. (2005). Aging and motivated cognition: the positivity effect in attention and memory. *Trends Cogn. Sci.* 9 496–502. 10.1016/j.tics.2005.08.005 16154382

[B63] Mather M. Knight M. R. (2005). Goal-directed memory: the role of cognitive control in older adults’ emotional memory. *Psychol. Aging* 20 554–570. 10.1037/0882-7974.20.4.554 16420131

[B64] McKee M. Stuckler D. (2001). The assault on universalism: how to destroy the welfare state. *BMJ* 343:7973. 10.1136/bmj.d7973 22187190

[B65] Mojtabai R. Jorm A. F. (2015). Trends in psychological distress, depressive episodes and mental health treatment-seeking in the United States: 2001-2012. *J. Affect. Disord.* 174 556–561. 10.1016/j.jad.2014.12.039 25556674

[B66] Mroczek D. K. Kolarz C. M. (1998). The effect of age on positive and negative affect: a developmental perspective on happiness. *J. Pers. Soc. Psychol.* 75 1333–1349. 10.1037/0022-3514.75.5.1333 9866191

[B67] Murphy N. A. Isaacowitz D. M. (2008). Preferences for emotional information in older and younger adults: a meta-analysis of memory and attention tasks. *Psychol. Aging* 23 263–286. 10.1037/0882-7974.23.2.263 18573002

[B68] Newsom J. T. Schulz R. (1996). Social support as a mediator in the relation between functional status and quality of life in older adults. *Psychol. Aging* 11 34–44. 10.1037/0882-7974.11.1.34 8726368

[B69] Niemann H. Sturm W. Thöne-Otto A. Willmes-von-Hinkeldey K. (2008). *CVLT- California Verbal Learning Test - German Adaption.* Frankfurt: Pearson Clinical &Talent Assessment.

[B70] Ochsner K. N. Gross J. J. (2005). The cognitive control of emotion. *Trends Cogn. Sci.* 9 242–249. 10.1016/j.tics.2005.03.010 15866151

[B71] Petrides K. V. Furnham A. (2006). The role of trait emotional intelligence in a gender-specific model of organizational variables. *J. Appl. Soc. Psychol.* 36 552–569. 10.1111/j.0021-9029.2006.00019.x

[B72] Powers C. Wisocki P. Whitbourne S. (1992). Age differences and correlates of worrying in young and elderly adults. *Gerontologist* 32 82–88. 10.1093/geront/32.1.82 1740260

[B73] Quigley L. Nelson A. L. Carriere J. Smilek D. Purdon C. (2012). The effects of trait and state anxiety on attention to emotional images: an eye-tracking study. *Cogn. Emot.* 26 1390–1411. 10.1080/02699931.2012.662892 22646929

[B74] R Development Core Team (2008). *R: A Language and Environment for Statistical Computing.* Vienna: R Foundation for Statistical Computing http://www.R-project.org

[B75] Reed A. E. Carstensen L. L. (2012). The theory behind the age-related positivity effect. *Front. Psychol.* 3:339. 10.3389/fpsyg.2012.00339 23060825 PMC3459016

[B76] Reed A. E. Chan L. Mikels J. A. (2014). Meta-analysis of the age-related positivity effect: age differences in preferences for positive over negative information. *Psychol. Aging* 29 1–15. 10.1037/a0035194 24660792

[B77] Reitan R. M. (1992). *Trail Making Test. Manual for Administration and Scoring.* Tucson, AZ: Reitan Neuropsychology Laboratory.

[B78] Revelle W. Rocklin T. (1979). Very simple structure: an alternative procedure for estimating the optimal number of interpretable factors. *Multivar. Behav. Res.* 14 403–414. 10.1207/s15327906mbr1404_2 http://personality-project.org/revelle/publications/vss.pdf 26804437

[B79] Russell D. W. Cutrona C. E. (1991). Social support, stress, and depressive symptoms among the elderly: test of a process model. *Psychol. Aging* 6 190–201. 10.1037/0882-7974.6.2.190 1863388

[B80] Scheibe S. Sheppes G. Staudinger U. M. (2015). Distract or reappraise? Age-related differences in emotion-regulation choice. *Emotion* 15 677–681. 10.1037/a0039246 25961143

[B81] Schulz P. Schlotz W. (1999). Trierer Inventar zur Erfassung von chronischem Stress (TICS): Skalenkonstruktion, teststatistische Überprüfung und Validierung der Skala Arbeitsüberlastung. *Diagnostica* 45 8–19. 10.1026//0012-1924.45.1.8

[B82] Siegling A. B. Vesely A. K. Petrides K. V. Saklofske D. H. (2015). Incremental Validity of the Trait Emotional Intelligence Questionnaire – Short Form (TEIQue–SF). *J. Pers. Assess.* 97 525–535. 10.1080/00223891.2015.1013219 25830494

[B83] Simon J. R. Small A. M. Jr. (1969). Processing auditory information: interference from an irrelevant cue. *J. Appl. Psychol.* 53 433–435. 10.1037/h0028034 5366316

[B84] Stawski R. S. Sliwinski M. J. Almeida D. M. Smyth J. M. (2008). Reported exposure and emotional reactivity to daily stressors: the roles of adult age and global perceived stress. *Psychol. Aging* 23 52–61. 10.1037/0882-7974.23.1.52 18361654 PMC3485068

[B85] Streubel B. Kunzmann U. (2011). Age differences in emotional reactions: arousal and age-relevance count. *Psychol. Aging* 26 966–978. 10.1037/a0023424 21517185

[B86] Sullivan S. Ruffman T. Hutton S. (2007). Age differences in emotion recognition skills and the visual scanning of emotion faces. *J. Gerontol.* 62B 53–60. 10.1093/geronb/62.1.p53 17284558

[B87] Sutin A. R. Terracciano A. Milaneschi Y. An Y. Ferrucci L. Zonderman A. B. (2013). The effect of birth cohort on well-being: the legacy of economic hard times. *Psychol. Sci.* 24 379–385. 10.1177/0956797612459658 23349030 PMC3602296

[B88] Sweeting H. West P. Young R. Der G. (2010). Can we explain increases in young people’s psychological distress over time? *Soc. Sci. Med.* 71 1819–1830. 10.1016/j.socscimed.2010.08.012 20870334 PMC2981856

[B89] Thompson L. A. (1995). Encoding and memory for visible speech and gestures: a comparison between young and older adults. *Psychol. Aging* 10 215–228. 10.1037/0882-7974.10.2.215 7662181

[B90] Vazquez C. Blanco I. Sanchez A. McNally R. J. (2016). Attentional bias modification in depression through gaze contingencies and regulatory control using a new eye-tracking intervention paradigm: study protocol for a placebo-controlled trial. *BMC Psychiatry* 16:439. 10.1186/s12888-016-1150-9 27931196 PMC5146883

[B91] Velicer W. (1976). Determining the number of components from the matrix of partial correlations. *Psychometrika* 41 321–327. 10.1007/bf02293557

[B92] Westerhof G. J. Keyes C. L. M. (2006). After the fall of the Berlin Wall: perceptions and consequences of stability and change among middle-aged and older East and West Germans. *J. Gerontol. Ser. B Psychol. Sci. Soc. Sci.* 61B 240–247. 10.1093/geronb/61.5.S240 16960237

[B93] Ziaei M. von Hippel W. Henry J. D. Becker S. I. (2015). Are age effects in positivity influenced by the valence of distractors? *PLoS One* 10:e0137604. 10.1371/journal.pone.0137604 26366872 PMC4569566

[B94] Zimmermann P. Fimm B. (2012). *TAP. Testbatterie zur Aufmerksamkeitsprüfung Version* 2.3. Herzogenrath: Psychologische Test.

