# Supplementary material for: Positivity in Younger and in Older Age: Associations With Future Time Perspective and Socioemotional Functioning
Source: Front Psychol. 2020 Nov 17;11:567133. doi: 10.3389/fpsyg.2020.567133 (PMC7705101; doi:10.3389/fpsyg.2020.567133)
Supplement: Supplementary file 1 [file Table_1.DOCX]

Supplementary Material

# Supplementary Data

**Follow-up Analyses.** Our results showed that age moderates the relationship between FTP and positivity. We ran a follow-up analysis to test whether differences in well-being levels differently predict the relationship between FTP and the positivity shift in younger and in older adults. Positivity shift scores were regressed onto FTP, Age group, and Well-being, and all interactions among Age group, FTP, and Well-being. FTP had a significant negative main effect on the positivity shift, (*b* = .46, *SEb* = .13, *β* = .67, *p* = .041). The interactions between Age Group X FTP, (*b* = -.63, *SEb* = .17, *β* = -.61, *p* < .001, see Figure 2), and between Age Group X Well-being X FTP, (*b* = .05, *SEb* = .02, *β* = .38, *p* = .043) were significant. We probed this 3-way interaction testing the effect of FTP on the positivity shift for lower and higher (the mean ±1 SD) levels of well-being separately for the two age groups. In younger adults, FTP showed a significant main effect, (*b* = .48, *SEb* = .14, *β* = .47, *p* < .001). The interaction between FTP X Well-being was not significant (*p* = .201). In older adults, FTP showed a non-significant trend, (*b* = -.18, *SEb* = .09, *β* = -.25, *p* = .057), and an interaction between Well-being X FTP that approached significance, (*b* = .02, *SEb* = .01, *β* = .28, *p* = .051). The negative relationship between FTP and positivity indicated older adults with lower well-being levels (See Supplementary Figure 1).

# Supplementary Figures


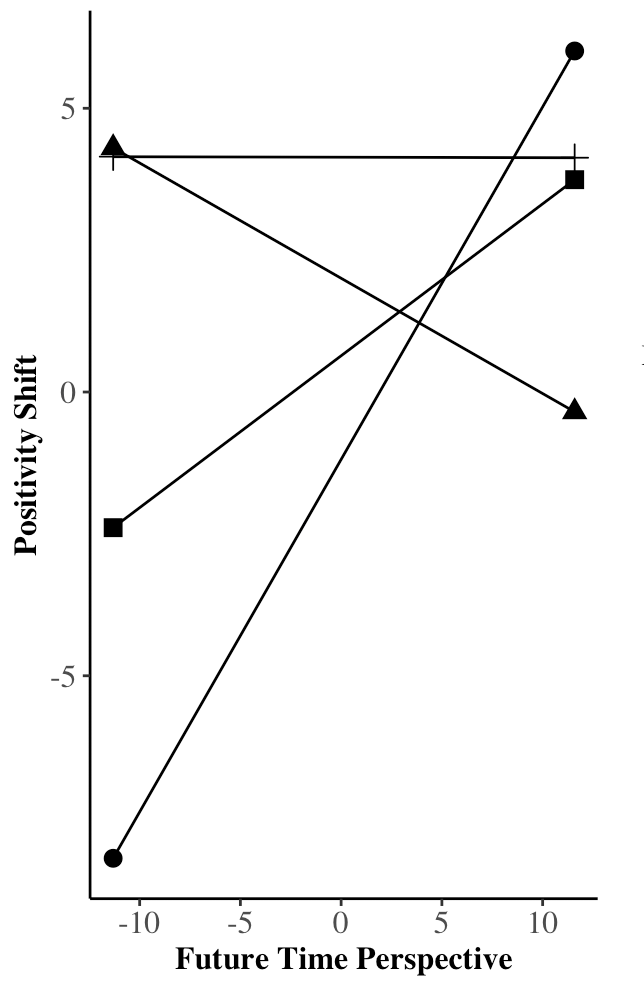

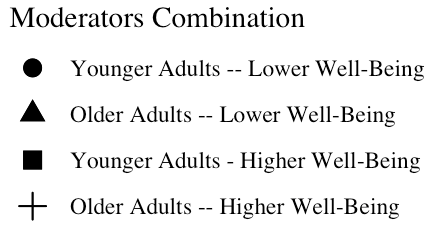


Supplementary Figure 1. Positivity shift as a function of age, well-being, and FTP levels. Lines are regression slopes from simple slope analysis for 1 SD below the mean, and 1 SD above the mean well-being levels for younger and older adults.
